# RNA–Protein Interactions Prevent Long RNA Duplex Formation: Implications for the Design of RNA-Based Therapeutics

**DOI:** 10.3390/molecules23123329

**Published:** 2018-12-15

**Authors:** Eckart Bindewald, Lisheng Dai, Wojciech K. Kasprzak, Taejin Kim, Shuo Gu, Bruce A. Shapiro

**Affiliations:** 1 RNA Biology Laboratory, Basic Science Program, Frederick National Laboratory for Cancer Research supported by the National Cancer Institute, Frederick, MD 21702, USA; eckart@mail.nih.gov (E.B.); kasprzaw@mail.nih.gov (W.K.K.); 2 RNA Biology Laboratory, National Cancer Institute, Frederick, MD 21702, USA; lisheng.dai@nih.gov (L.D.); taejin.kim@nih.gov (T.K.)

**Keywords:** RNA–RNA, interaction, RNA-binding, RNA-binding protein (RBP), ribosomal RNA (rRNA), Dicer, helix

## Abstract

Cells frequently simultaneously express RNAs and cognate antisense transcripts without necessarily leading to the formation of RNA duplexes. Here, we present a novel transcriptome-wide experimental approach to ascertain the presence of accessible double-stranded RNA structures based on sequencing of RNA fragments longer than 18 nucleotides that were not degraded by single-strand cutting nucleases. We applied this approach to four different cell lines with respect to three different treatments (native cell lysate, removal of proteins, and removal of ribosomal RNA and proteins). We found that long accessible RNA duplexes were largely absent in native cell lysates, while the number of RNA duplexes was dramatically higher when proteins were removed. The majority of RNA duplexes involved ribosomal transcripts. The duplex formation between different non-ribosomal transcripts appears to be largely of a stochastic nature. These results suggest that cells are—via RNA-binding proteins—mostly devoid of long RNA duplexes, leading to low “noise” in the molecular patterns that are utilized by the innate immune system. These findings have implications for the design of RNA interference (RNAi)-based therapeutics by imposing structural constraints on designed RNA complexes that are intended to have specific properties with respect to Dicer cleavage and target gene downregulation.

## 1. Introduction

RNA–RNA interactions play structural and functional roles in nearly every step of the gene expression pathway, such as RNA interference/microRNA pathways, RNA editing, RNA splicing, and interferon response. A variety of approaches were developed to map RNA sequencing (RNA-Seq) data to the genome, such as Hierarchical Indexing for Spliced Alignment of Transcripts (HISAT) [1] and Shape-Seq [2]. RNA–RNA interactions that are associated with a common protein-binding partner can be ascertained using the cross-linking, ligation, sequencing of hybrids (CLASH) approach [3]. Affinity purification is used to obtain specific proteins that potentially bind different RNAs. A ligation step leads to RNA–RNA chimeras, provided they are in spatial proximity. This technique was used to determine the microRNA–messenger RNA (mRNA) interactome [4].

In addition, RNA antisense purification (RAP) can be used to determine RNA–DNA interactions [5]. An RNA-centric version (RNA-RAP) consisting of cross-linking, and RNA capture with antisense oligonucleotides followed by RNA sequencing was developed and utilized to determine interactions of U1 small nuclear RNA (snRNA), as well as Malat1 long non-coding RNA (lncRNA) [6]. RNA proximity ligation (RPL) was utilized to identify intra- and possibly inter-molecular RNA–RNA interactions genome-wide [7]. Interestingly, it was found that the data are dominated by ribosomal intra-molecular data. The data were not strongly supportive of inter-molecular trans-RNA–RNA interactions. Furthermore, a method for determining sequence specificity of RNA–protein interactions based on deep sequencing data was reported recently [8]. A list of protein–RNA interaction databases was recently published [9], comprising the DataBase of RNA-Binding Protein specificities (RBPDB) [10], the Catalog of Inferred Sequence Binding Preferences of RNA binding proteins (CISBP-RNA) [11], RNA interaction networks from large-scale CLIP-Seq data (starBase) [12], doRiNa [13] and a database of experimentally determined binding sites of RNA-binding proteins (CLIPz) [14].

The length of potential RNA–RNA duplexes influences their role in the formation of ribonucleoprotein (RNP) granules. Recently, a phase-transition model was proposed where, depending on the local concentrations of RNAs and proteins, RNP granule formation is either protein-driven, RNA-driven, or driven by both RNA–RNA and protein–protein interactions [15]. The emphasis of this model is on RNA–RNA interactions mediated by RNA–RNA binding sites that occur due to chance with a higher likelihood for long RNAs.

It is also known that a variety of cellular surveillance mechanisms are sensitive to the length of RNA duplexes. In addition to the aforementioned RNA interference (RNAi) pathway that is triggered by ~21-bp helices, the RNA-binding protein, protein kinase R (PKR), is activated by interactions with long RNA helices consisting of at least 30 base pairs, which then triggers downstream effects such as downregulation of protein synthesis or even apoptosis [16,17]. Exogenous RNA helices with more than 40 base pairs can trigger an inflammatory response via Toll-like receptor 3 (TLR3). In contrast, the protein melanoma differentiation-associated gene 5 (MDA5) binds to very long double-stranded RNA (>1000 bp) and can subsequently promote filament formation and innate immune activation [18].

Finally, the formation of long duplexes via RNA–RNA interactions has potential therapeutic applications. For example, self-assembling RNA complexes that contain functional Dicer-substrate RNA helices were utilized for target gene downregulation. Such designed RNA complexes may correspond to quasi-symmetric nanostructures resembling rings [19,20], but other reported RNA nanostructures like RNA cubes may potentially be functionalized in a similar fashion [21]. Another approach for designed RNA complexes are RNA switches that change to a functional conformation in the presence of another RNA that acts as biomarker and trigger. Such conditionally activated RNA switches may for example expose upon activation a long RNA helix that can then be processed by Dicer, ultimately leading to the downregulation of a target gene [22]. All these findings highlight the importance in studying RNA–RNA interactions, in particular the formation of long RNA duplexes, in the cellular environment. However, despite the efforts described above in characterizing the RNA–RNA interactome, it is still unclear whether long helices resulting from extended RNA–RNA base-pairing exist in cells.

Here, we present a novel method for enriching and cloning cellular double-stranded RNAs (dsRNAs) in their physiological condition. The idea of this approach is to utilize RNA-Seq combined with RNA single-strand cutting enzymes in order to obtain sequencing information pertaining to long RNA duplexes. Furthermore, a variety of bioinformatics analysis approaches were utilized in order to computationally characterize RNA–RNA interactions. Applying such methods, we investigated whether or not long RNA–RNA base pairs with more than 18 base pairs are formed in human cells.

## 2. Results

### 2.1. Digestion of Non-Perfect RNA Duplexes with Endoribonucleases

**Figure 1:**
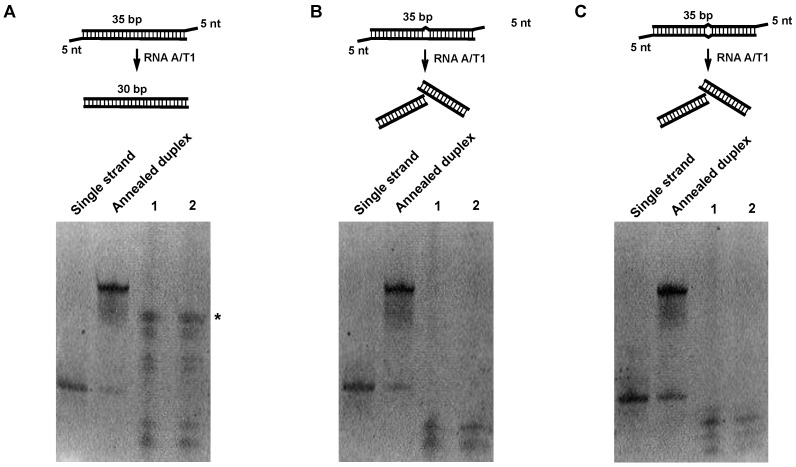
Digestion of RNA duplex oligos with RNAse A/T1. Initially, 1 μg of each RNA duplex oligo was digested with different concentrations of RNAse A/T1 at 37 °C for 30 min. RNA was separated on 15% acrylamide Tris/borate/ethylenediaminetetraacetic acid (TBE) native gels and stained with SYBR gold. (**A**) Digestion of perfect matched RNA duplex; * indicates an enriched RNA duplex. (**B**) Digestion of RNA duplex with bulge. (**C**) Digestion of RNA duplex with internal loop. Lane 1, 1:200 dilutions of RNAse A/T1 cocktail. Lane 2, 1:500 dilutions of RNAse A/T1 cocktail.

To enrich RNA duplexes, we sought to digest single-stranded RNAs (ssRNAs) using endoribonucleases. Given that the combined formulation of RNase A and RNase T1 is well characterized for specific digestion of single-stranded RNAs, a cocktail containing A and T1 (RNase A/T1), which is a standard, and a readily commercially available mixture, was chosen for the following experiments. A 35-bp-long dsRNA helix with five-nucleotide ssRNA as an overhang at both ends and two variants embedding a bulge or a mismatch in the middle was used as a control to probe the activity of RNase. The concentration of RNase A/T1, digestion time, and digestion temperature were optimized (see Section 4 for details). As shown in Figure 1, the RNA duplex with a bulge or mismatch was broken down while the perfect RNA duplex was preserved with only the overhang regions clipped off. We reason that such treatment will result in an enrichment of near-perfect RNA duplexes.

### 2.2. Enrichment of the Native Form of RNA Duplexes in Human Cells

**Figure 2:**
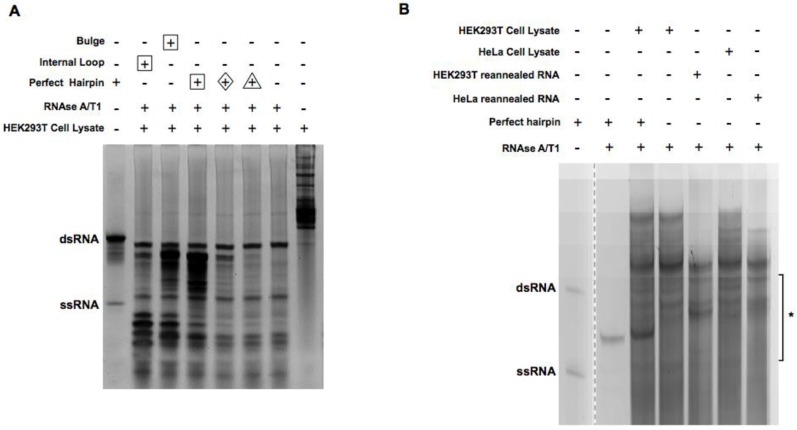
Digestion of cell lysate and reannealed RNA with RNAse A/T1. (**A**) HEK293T cell lysate spiked with different kinds of RNA duplex oligos were digested with 1:200 dilution of RNAse A/T1 at 37 °C for 30 min. RNA was extracted after digestion and separated on 15% acrylamide TBE native gels and stained with SYBR gold. Lanes with different amounts of spike-in RNA were labeled with different shapes (Δ, 0.1 μg; ◊, 0.5 μg; ☐, 5 μg). The arrow indicates the band that was subject to more detailed investigation as described in Section 2.3. (**B**) Cell lysate and reannealed RNA with or without spiked-in were digested with 1:200 dilution of RNAse A/T1. * indicates where the bands were cut for cloning and sequencing.

We sought to enrich dsRNA duplexes from human cells with the optimized digestion conditions. To keep the native form of RNA duplexes, HEK293T cell lysates were used directly for digestion. To track the efficiency of digestion and the enrichment of intended dsRNA duplexes synthetic RNA oligos and duplexes with known sequence/structure (Figure 1) were used as spike-in in the HEK293T cell lysate. Digestion was monitored on the gel after isolation of total RNA. As shown in Figure 2A, ssRNA spike-in was completely digested by RNase A/T1, while a perfectly paired control RNA duplex was preserved. Interestingly, RNA duplexes with non-perfect pairs in the middle were only partially removed, indicating RNase A/T1 activity was reduced in cell lysate, most likely due to the excess amount of cellular RNAs. Following the same conditions, we captured RNA duplexes in their native form from four cell lines (HEK293T, HeLa, MCF-7, and U2OS; labeled as native in Table S1, Supplementary Materials).

In parallel, we removed cellular proteins by extracting total RNAs from these four cell lines directly. The resulting RNAs, after ribosomal RNA (rRNA) removal, were heated up and reannealed before being subjected to the same RNase A/T1 digestion. In addition, total RNAs from HEK293T cells and HeLa cells were digested by RNAase A/T1 directly without the depletion of rRNA in order to capture RNA duplexes involving rRNAs. Samples with rRNA removal are labeled as “ribo-removed” while the ones without are labeled as “protein-removed” in Table S1 (Supplementary Materials). For all samples, RNA duplexes between 18 and 50 bp were enriched by gel extraction (Figure 2B), denatured, and then cloned with a previously reported method [23]. Their sequences were revealed by Illumina deep sequencing.

### 2.3. Focus on Pronounced Band

We created two additional sequencing datasets corresponding to HeLa and 293T cells, focusing on reads with molecular weight corresponding to a consistently exhibited band (indicated by an arrow in Figure 2a; also listed in Table S1, Supplementary Materials). We found that both datasets were dominated by one read with nucleotide sequence CGGGCGGCGGCGGUCGGCGGGC. This read could be mapped to expansion segment 27 of the 28S large ribosomal subunit (RefSeq identifier NR_003287, positions 3389–3410). This ribosomal segment is known to be involved in interactions with other cellular RNA [24].

A bioinformatics analysis of potential interactions of this sequence fragment with the transcriptome suggests that many cellular transcripts are subject to positive or negative selection for complementarity to this one ribosomal sequence region. We utilized the RNAduplex program to compute potential RNA–RNA interactions (including sub-optimal interactions within 2 kcal/mol with respect to the minimum free energy structure). The sum of the interaction energies of the minimum free energy structure, as well as suboptimal structures within 2 kcal/mol, was used as a score to measure the RNA–RNA interaction quantitatively. For each RNA of a certain class, a *z*-score was computed by comparing the interaction energy with respect to the native read sequence (the dominant read) to 20 interaction energies corresponding to shuffled versions of the same sequence. The resulting *z*-scores corresponding to each transcript are plotted in Figure S1 (Supplementary Materials). One can see that the complementarity to this rRNA fragment was over- or under-represented for many RNAs (corresponding to negative or positive *z*-scores for the interaction energy of the native rRNA sequence, respectively). The median of the *z*-scores was positive for most RNA types; in other words, on average, most RNAs seem to avoid interaction with this particular ribosomal RNA region. However, a functional analysis of the RNAs that have an overrepresentation of sites complementary to the 28S expansion segment (by choosing all transcripts that correspond to a *z*-score less than −3; other negative cut-offs led to similar results) showed an enrichment for genes that are associated with cellular compartments including the cell membrane, nuclear membrane, or the cytoskeleton.

### 2.4. The Effect of Removing Proteins

**Figure 3:**
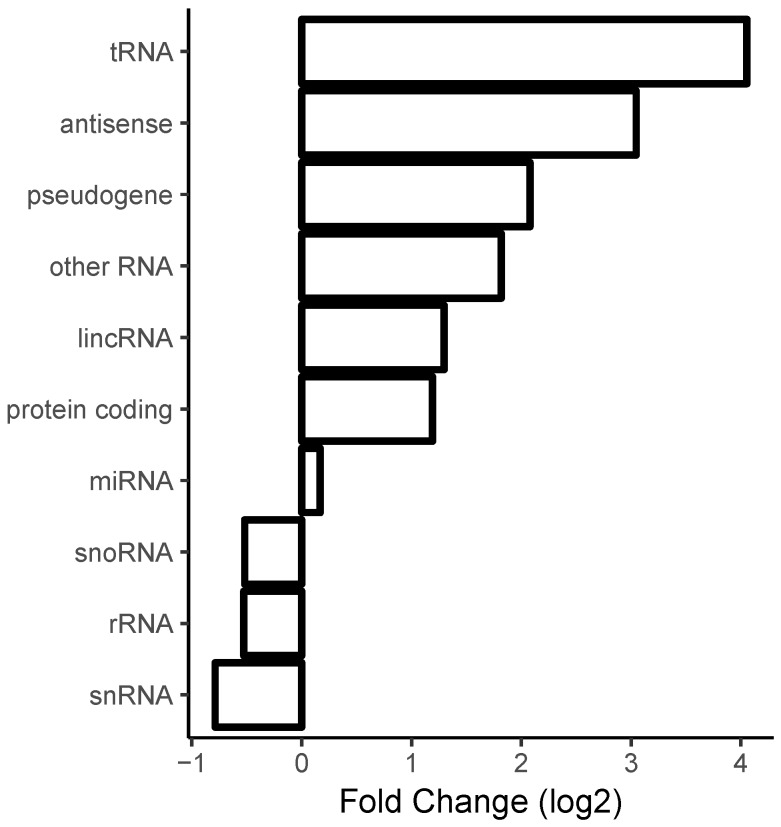
Enrichment of formation of long contiguous duplexes with respect to the removal of proteins. Upon the removal of proteins, most RNA classes such as transfer RNA (tRNA), antisense RNAs, messenger RNAs (mRNAs), and long intergenic non-coding RNA (lincRNA) genes were more likely to exhibit duplex formation, while ribosomal RNAs tended to be less structured. The gene class nomenclature follows the gene classification provided by ENSEMBL with the exception that a variety of subtypes of pseudogenes were combined into one “pseudogene” class.

We computed, for each gene, the differential expression (i.e., fold-changes) with respect to a particular treatment using (as described in Section 4) the alignment program Salmon followed by differential gene expression analysis via the R package DESeq2. Next, we averaged the values by RNA type (using a simplified version of the RNA type classification provided by ENSEMBL, see Section 4). The averaged fold-change values of the protein-removed datasets versus the lysate datasets is shown in Figure 3. One can see that transfer RNA (tRNA), antisense, long intergenic non-coding RNA (lincRNA), and mRNA transcripts were overrepresented and ribosomal RNA was underrepresented in the datasets corresponding to removal of proteins with respect to datasets corresponding to native lysate.

### 2.5. Correlation with Sense/Antisense Pairing

For many transcripts, one can identify regions that overlap with *cis*-antisense transcripts. We wanted to find out if regions corresponding to genomic overlaps between known transcripts with opposing strand orientation were more likely to correspond to regions to which reads were aligned (as described in Section 4, the HISAT program was used for mapping reads to the human genome assembly hg38). Each transcript has two possibilities of overlapping or not overlapping with mapped reads and another two possibilities of overlapping or not overlapping with another transcript of opposing strand directionality. This corresponds to 2 × 2 = 4 possible outcomes per transcript that can be tallied via contingency tables. Such contingency tables were created for each of the 13 datasets S1–S13 (shown in Table S2, Supplementary Materials). We found that, averaged over all 13 datasets, genes that are involved in *cis*-sense/antisense overlaps were, by a factor of 2.43 ± 0.35, more likely to have matches with respect to our duplex sequencing data. In other words, some fraction of cellular duplex formation can likely be attributed to simultaneous expression of *cis*-sense/antisense pairs. The magnitude of this effect was similar with respect to the three different experimental treatments (native lysates, protein removed, protein removed + ribosomal RNA removed).

### 2.6. Evidence for Endogenous Ligation Events

We searched for reads that partially matched two different transcripts. We required that the 5′ end of a read matched for at least 18 nucleotides (nt) with respect to one transcript, while the 3′ end of the same read matched another transcript (again for at least 18 nt). Also, reads that matched a transcript in their entirety or that could be explained by intra-transcript interactions were removed. Using this approach, we found very few cases of sufficient evidence for RNA ligation events.

### 2.7. Correlation with Solvent Accessibility in the Ribosome

**Figure 4:**
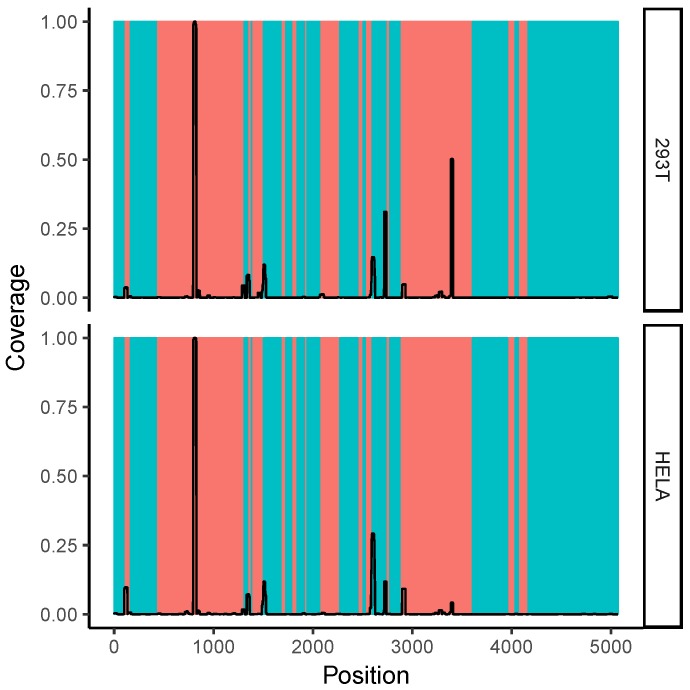
Read coverage with respect to 28S ribosomal RNA. Regions with orange background correspond to ribosomal expansion regions. One can see that regions with high read coverage are located within or adjacent to expansion regions. The shown data correspond to gel extraction of 293T cells or Hela cells (datasets S14 and S15 as listed in Table S1, Supplementary Materials). The coverage values were normalized such that, for each dataset, their highest coverage value on the 28S ribosomal RNA (rRNA) sequence was 1.0.

We mapped the per-nucleotide coverage of the obtained reads onto the 28S rRNA sequence. Shown in Figure 4 is the read coverage with respect to the complete sequence of 28S rRNA. One can see several distinct peaks of read coverage, all of which are in or adjacent to ribosomal expansion segments (shown in orange). Expansion segments are regions of the ribosome that are characterized by a large amount of structural flexibility [25] and are thought to participate in RNA–RNA and RNA–protein interactions [26,27]. A region around 28S rRNA expansion segment ES27L is shown in Figure S2B (Supplementary Materials), where we find a prevalence for one read (positions 3389–3410), but not the cognate region of the hairpin (positions 3445–3463). As elaborated on in Section 3, this region corresponds to a very high GC content and might have unique structural properties.

### 2.8. All-versus-All Search for Duplex Formation

**Figure 5:**
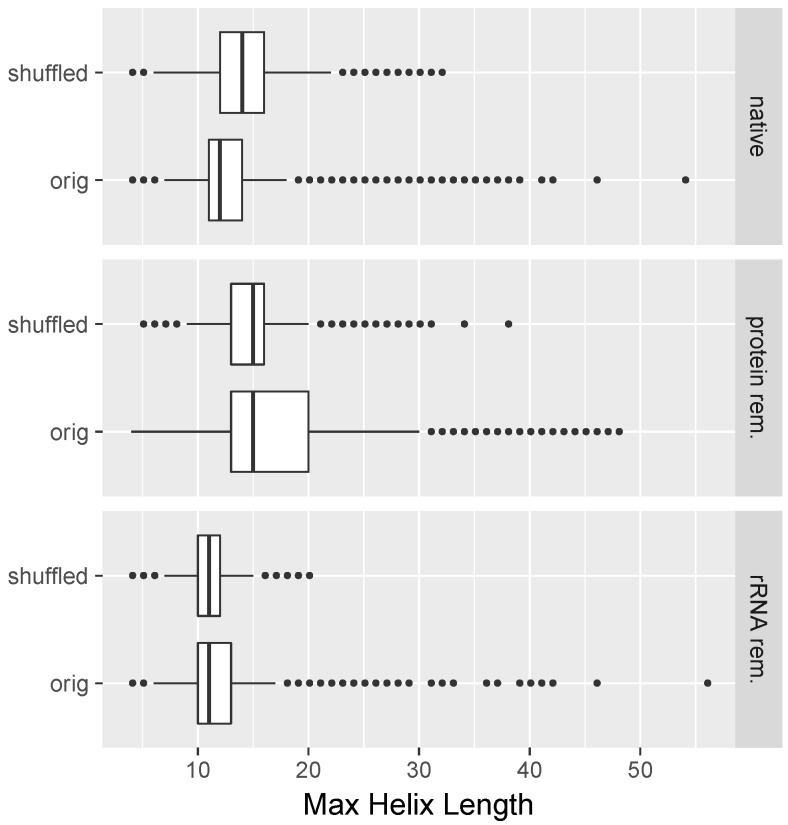
Evidence that co-expressed sequences are under constraints to avoid non-specific RNA–RNA interactions. Compared are shuffled and unshuffled read sequences. The maximum helix length read–read interactions for any given read (as predicted from RNAduplex) are plotted for different treatments. One can see that, for native cell lysates, shuffled sequences led to longer maximum contiguous helix lengths compared to the unshuffled case. For protein-removed datasets, the maximum helix lengths were longer and no substantial differences between shuffled and unshuffled sequences were observed.

We searched computationally in an all-versus-all manner for candidate read pairs of RNA–RNA duplex formation as described in Section 4. In Figure 5, we plotted, for each read, the longest helix that was found when running the structure prediction program (RNAduplex) with respect to all other reads of the same length within the same dataset. One can see that the majority of the longest found helices were for the dataset corresponding to native lysates mostly shorter than 14 base pairs long, and were virtually never longer than 18 base pairs with the exception of outliers (Figure 5, native/orig). Importantly, the median of the lengths of the longest found helices was increased when the read sequences were shuffled prior to performing the interaction prediction (Figure 5, native/shuffled). This restriction in maximum length of detected potential RNA helices was, to a large extent, lifted in the case of the protein-removed datasets; with the exception of outliers, the maximum lengths of found helices was now 30 base pairs. Furthermore, comparison with shuffled sequences (Figure 5, protein rem./shuffled) suggests that this value of 30 base pairs is greater than what one would expect by chance. For both the protein-removed and ribosome-removed datasets, there was no pronounced difference in the median of predicted helix lengths between their respective shuffled and unshuffled versions.

## 3. Discussion

In this paper we obtained transcriptome-wide information about long RNA duplexes by utilizing RNA-Seq and RNA single-strand processing enzymes on the experimental side followed by a variety of bioinformatics analyses. We arrived at interesting conclusions that are discussed below.

### 3.1. Long Perfectly Complementary Regions Are Relatively Uncommon

The “pan-transcriptome” (the set of all known transcripts, not necessarily expressed simultaneously) contains many *cis* and *trans* sense/antisense regions. It was found that cells frequently express sense and antisense transcripts simultaneously [28]. The lack of apparent long RNA duplex regions in cell lysates suggests that many RNAs are hindered from undergoing extensive inter- and intra-molecular interactions via mechanisms other than nucleotide base-pairing. A variety of mechanisms may contribute to this lack of detectable RNA duplexes. A strong bias toward short helices with fewer than 12–14 base pairs was also reported as part of an analysis of RNAs with known three-dimensional (3D) structure [29]. In order to avoid a bias of experimental observations in the form of intra-strand interactions that consist of short helices, the work presented here focused on helices with more than 18 base pairs.

We found, in the present experimental results, orders of magnitude higher occurrence of duplexes in cell extracts where proteins were removed compared to cell lysates. This suggests that RNA-binding proteins prevent a large portion of RNA–RNA interactions. Moreover, the difference with respect to the presence of ribosomal RNA in observed evidence for RNA–RNA interactions for datasets where proteins were removed suggests that one important function of RNA-binding proteins is the prevention of interactions between ribosomal and non-ribosomal RNAs.

Also, the found difference in medium helix length in an all-versus-all search of all read pairs in native lysates (with shuffled sequences leading to longer predicted duplexes compared to unshuffled read sequences) suggests that there is a pervasive cellular constraint on preventing the formation of long duplexes. As described, we computed a duplex structure for all reads with respect to all other reads of the same length in the same dataset. One can, in this way, for any read, identify the length of the longest predicted helix. We then computed, for each dataset, the median of the longest predicted helices. We found that the median of the helix length with the longest predicted interaction duplex was only 12 base pairs (Figure 5, native/orig). The fact that shuffled reads correspond to longer predicted helix lengths further underscores that the prevention of the formation of long uninterrupted helices is an active, important, and non-coincidental constraint on the RNA transcriptome. An avoidance of cognate sequences that could lead to stochastic interactions was reported by Umu et al. [30].

Indeed, we found the lack of detectable potential helix formation in the native lysate datasets rather striking. On the other hand, the in vitro experiments indicate that the enzymes cut strands of helices that contain single-base bulges or internal loops. Given that, one would expect for a dataset consisting of reads longer than 18 nt treated as described for the native lysate datasets to exhibit a plethora of potential RNA duplexes consisting of more than 18 contiguous base pairs. Yet, this was not at all what we found when analyzing the experimental data; as mentioned, we found a striking lack of long helices (we found a median length of 12 base pairs and virtually never predicted helices longer than 18 base pairs for the native lysate datasets). What would explain this striking discrepancy between expectation and obtained experimental results? As possible explanations, we propose (given the premise that extensive RNA–RNA interactions exist, but they rarely contain long helices) the following: (a) reads corresponding to mismatch-containing helices were found in our datasets because they were protected from degradation by proteins at the stage of enzyme digestion of single-stranded regions; (b) reads corresponding to mismatch-containing helices were found in our datasets because the degradation by single-strand cleaving enzymes was incomplete. The fact that we found an increase in predicted contiguous helix lengths in the case of the protein-removed datasets, combined with the fact that, for all datasets, we found a large fraction of reads (at least 50%) for which the longest predicted helix interaction could not account for the entire read length, suggests that both scenarios (a) and (b) contribute to the observed findings.

Furthermore, duplexes longer than about 18 base pairs can be processed by Dicer, leading to the shortening of duplexes and the degradation of the constituent transcripts. Dicer-processed duplexes were reported, in some cases, to result in endo small interfering (RNA) that then can lead to post-transcriptional downregulation of mRNAs [31].

Long RNA duplexes correspond to a danger- or pathogen-associated molecular pattern (DAMP or PAMP), that may trigger a response from the innate immune system via several pathways. Duplexes with lengths greater than 30 base pairs can trigger a global cellular response of downregulation of translation or even apoptosis via binding to protein kinase R (PKR). The 2,5-AS/RNA L pathway is triggered by RNA helices with lengths of about 70 base pairs or longer. This pathway—thought to be an innate immune response to invading viral RNA—leads to the degradation of the recognized RNA. It should, thus, be no surprise that long duplexes are uncommon in cells because long duplexes are, as indicated, targeted for degradation by several different kinds of endo-nucleases. Also, the DAMP/PAMP “danger” pattern would be less informative and more difficult to interpret by the cell if there were a substantial amount of “beneficial” long RNA duplexes. In other words, our experimental results fit very well into the current thought regarding the workings of the innate immune system.

Additionally, we searched for chimeric reads that mapped to two distinct transcript sequences. Those reads could be evidence for endo-ligase events. With the exception of ribosomal RNA, we did not find chimeric reads that would consistently support particular RNA–RNA interactions. The lack of perfectly complementary regions and endo-ligation events suggests that the majority of the observed sequence data are a result of partial digestion by the administered nuclease cocktail or protein-covered single-stranded regions. This allows, nonetheless, the firm conclusion that long perfectly complementary RNA duplexes are virtually non-existent in the examined cell lines.

### 3.2. Ribosomal RNA Dominates RNA–RNA Interactions

We found that a large portion of reads mapped to ribosomal RNA. Also, a substantial fraction of reads mapped to ribosomal expansion segments. These are regions that are likely to be flexible and located near the surface of the ribosome structure or are protruding outward. This suggests that flexible regions at distal ribosomal regions are important for RNA–RNA interactions. One may note an analogy between weakly structured or unstructured RNA regions and unstructured regions of proteins that were frequently found to be important for inter-molecular binding and recognition events [32].

An extreme case of this tendency toward surface-accessible regions is the high coverage of 28S ribosomal expansion segments 7 (ES7L) and 27 (ES27L) in the analysis of the gel-extraction experiments (see Section 2). Interestingly, we found no prevalence of cognate reads that would form an extended duplex in the case of the ES27L region shown in Figure S2B (Supplementary Materials). This raises the question why the read was found in the dataset at all. It should be noted that the read in question is extremely GC rich (GC content 95.5%). It was suggested that ribosomal expansion segments may participate in triple-helix formation due to their high GC content [26,27]. The same authors suggest that one function of ribosomal expansion elements is related to the storage and retrieval of RNAs with respect to ribonucleoprotein complexes (RNPs). This role fits well with our observation that RNAs with pronounced complementarity to the 28S expansion segment 27 (ES27L) are enriched for cellular compartments including the cell membrane, nuclear membrane, or the cytoskeleton, because those correspond to typical localizations of RNPs [33].

It seems plausible that ribosomal segments whose role it is to be highly interacting with RNAs and proteins are both weakly folded and yet protected from single-strand cutting nucleases via unusual structural features such as triple helices. More research will be needed to elucidate the structural biology of ribosomal expansion segments.

We consistently found that the number of contiguous RNA duplexes was dramatically higher if proteins were removed. This suggests, that RNA-binding proteins—to a large extent—prevent RNA base-pairing. In other words, RNAs are more prone to be involved in inter-strand interactions in vitro compared to in vivo environments. In the case of intra-strand interactions, Rouskin and coworkers stated that there “are vastly fewer structured mRNA regions in vivo than in vitro” for the case of intra-strand interactions [34]. This suggests that one general function of proteins is to prevent non-specific RNA–RNA interactions. This begs the question why RNA–RNA interactions are not utilized more prevalently in cells. Well-known exceptions such as microRNA–mRNA interactions are “moderated” by a set of specialized proteins that are part of the RNAi pathway. We submit that non-specific RNA–RNA interactions may be potentially harmful because they could lead to uncontrolled run-away aggregation of RNA strands. Specific RNA–RNA interactions might have the potential disadvantage of being straightforward to mimic or disrupt by invading pathogens.

### 3.3. Implications for the Design of RNA-Based Therapeutics

A variety of strategies for utilizing RNA for therapeutic or diagnostic purposes were reported in the literature. These approaches are typically based on one of a handful of foundational technologies such as RNA interference (RNAi), clustered regularly interspaced short palindromic repeats (CRISPR), ribozymes, antimirs, or antisense RNAs. In all cases, the exogenously delivered RNAs are potentially subject to human cellular surveillance. This work highlights that an exogenous long RNA duplex can potentially be unambiguously recognized because intra-cellular occurrence of long RNA duplexes is apparently rare. A fitting metaphor might be that a “coin dropped into an empty jar makes a lot of noise”.

One of two opposing strategies for the design of RNA-based therapeutics present themselves; one obvious strategy is to avoid the utilization of long RNA duplexes in RNA complexes where stability and a long half-life is desired. Alternatively, purposefully introduced modified bases might increase half-life even for long RNA duplexes. A second strategy is to deliver an abundance of long RNA duplexes or activate conformational changes to induce duplex formation in target cells. In this case, cells might recognize a danger-associated molecular pattern and might trigger a response of innate immunity such as apoptosis. For example, the PKR protein can be activated by helices longer than ~30 base pairs, which can trigger downregulation of protein production (i.e., translation) or even cell death. This mechanism was utilized for the conditional activation of PKR-mediated cell death via a duplex consisting of the Bcr/Abl fusion oncogene (typical for chronic myelogenous leukemia) and an exogenous designed cognate antisense strand [35]. In light of such findings of induced cell death caused by a single type of long RNA duplex, it seems plausible that long endogenous RNA helices are rare or virtually absent in healthy cells (as we found in the work presented in this paper).

Designers of RNAi-based therapeutics should be aware of recent results related to the processivity of components of the RNAi pathway such as Dicer. The introduction of mismatches of a Dicer substrate RNA will likely reduce the effectiveness of its inhibition function. On the other hand, cleavage of a helical region by Dicer may not necessarily be prevented by a small number of mismatches or even bulges [36,37]. Taken together, designers of RNA structures need to be aware if their RNA-based constructs are above or below the “radar” of RNA-structure-based cellular surveillance.

## 4. Materials and Methods

### 4.1. Cell Culture

HEK293T, HeLa, U2OS, and MCF-7 cells were maintained in high-glucose Dulbecco’s modified Eagle’s medium (DMEM) (ThermoFisher Scientific, Waltham, MA, USA) containing 10% fetal bovine serum (FBS) (HyClone, Logan, UT, USA) and penicillin/streptomycin antibiotics (ThermoFisher Scientific, Waltham, MA, USA) at 37 °C and 5% CO_2_. Cell pellets were collected for the following experiments.

### 4.2. RNA Oligos

For the purposes of enzyme digestion optimization and spike-in, we synthesized four RNA oligos using partial green fluorescent protein (GFP) mRNA sequences (same or reverse complement) as templates. Sequence A (5′-rArGrCrArGrArArCrArCrCrCrCrCrArUrCrGrGrCrGrArCrGrGrCrCrCrCrGrUrGrCrUrG-3′) was annealed with B (5′-rCrGrGrGrGrCrCrGrUrCrGrCrCrGrArUrGrGrGrGrGrUrGrUrUrCrUrGrCrUrGrCrUrGrC-3′), C (5′-rCrGrGrGrGrCrCrGrUrCrGrCrCrGrArUrUrGrGrGrGrGrUrGrUrUrCrUrGrCrUrGrCrUrGrC-3′), or D (5′-rCrGrGrGrGrCrCrGrUrCrGrCrCrGrArUrArGrGrGrGrUrGrUrUrCrUrGrCrUrGrCrUrGrC-3′) to generate three kinds of different RNA duplexes (Integrated DNA Technologies, Coralville, IA, USA). Annealed A/B had 30 nt of perfect duplex with a 5-nt overhang at both 3′ ends, Annealed A/C contained one bulge in the middle of an RNA duplex, and annealed A/D contained an internal loop in the middle of an RNA duplex.

### 4.3. RNase A/T1 Digestion

RNase A/T1 cocktail (500 U/mL RNase A and 20,000 U/mL RNase T1) (ThermoFisher Scientific, Waltham, MA, USA) was used for enriching RNA duplexes. For optimization, 1 μg of annealed oligos were digested in 150 μL of digestion buffer (300 mM NaCl, 10 mM Tris–Cl, 5 mM ethylenediaminetetraacetic acid (EDTA), pH 7.4) with different concentrations of RNase A/T1 (1:100, 1:200, and 1:500) at 37 °C for 30 min. For optimizing the RNase A/T1 condition in the context of cellular RNA, 1 × 10^6^ cells were lysed on ice with 50 μL of radioimmunoprecipitation assay (RIPA) buffer (25 mM Tris-HCl pH 7.6, 150 mM NaCl, 1% NP-40, 1% sodium deoxycholate, 0.1% SDS) (ThermoFisher Scientific, Waltham, MA, USA). After adding different annealed RNA oligos as spike-in, cell lysates were digested in an additional 100 μL of digestion buffer with RNase A/T1 (1:200) at 37 °C for 30 min. RNA was separated on 15% acrylamide Tris/borate/EDTA (TBE) (K-D Medical, Columbia, MD, USA) native gels and stained with SYBR gold (ThermoFisher Scientific, Waltham, MA, USA). Images were obtained with Chemidoc Imaging system (Bio-Rad, Hercules, CA, USA).

### 4.4. Cellular RNA Duplex Enrichment

For enrichment of cellular RNA duplexes, three different methods were performed as described below. To monitor the sensitivity of the following experimental procedure, 50 copies per cell of perfect RNA oligo duplexes (0.085 fmol in total) were added into 1 × 10^6^ cell pellets before lysing the cells or extracting total RNA.

For digestion of native cell lysate, 1 × 10^6^ cells were lysed on ice with 50 μL of RIPA buffer. Cell lysates were digested in an additional 100 μL of digestion buffer with RNase A/T1 (1:200) at 37 °C for 30 min.

For removal of proteins and RNA reannealing, total RNA was extracted from cells with Trizol (ThermoFisher Scientific, Waltham, MA, USA) following the manufacturer’s instructions. After heating at 95 °C for 3 min, total RNAs were hybridized at 42 °C overnight in 10 μL of hybridization buffer (40 mM piperazine-*N*,*N*′-bis(2-ethanesulfonic acid) (PIPES), 1mM EDTA, 400 mM NaCl, 80% formamide, pH 6.4) (Ambion, Austin, TX, USA). The reannealed RNA was digested in 150 μL of digestion buffer at 37 °C for 30 min.

For removal of ribosomal RNA, total RNA was treated with Ribo-Zero™ Magnetic Gold Kit (Epicentre, Charlotte, NC, USA) following the manufacturer’s instructions. After heating at 95 °C for 3 min, treated RNAs were hybridized at 42 °C overnight in 10 μL of hybridization buffer. The reannealed RNA was digested in 150 μL of digestion buffer at 37 °C for 30 min.

Digested RNA from cell lysate or reannealed RNA was extracted with acid phenol/chloroform (Ambion, Austin, TX, USA) following the manufacturer’s instructions. After treatment with a DNAse I kit (Ambion, Austin, TX, USA), RNA was separated on 15% acrylamide TBE native gels and stained with SYBR gold (ThermoFisher Scientific). Images were obtained with Chemidoc Imaging system (Bio-Rad, Hercules, CA, USA).

### 4.5. Cloning of dsRNAs and Deep Sequencing

#### 4.5.1. Gel Extraction

The digested RNA products were separated on 15% acrylamide TBE native gels. RNA duplexes around 18–50 bp were sliced from gels and broken down by gel breaker tubes (IST Engineering, Milpitas, CA, USA). RNAs were eluted overnight at 4 °C, and gel debris were filtered out by 5-μm filter tubes (IST Engineering). Eluted RNAs were precipitated by ethanol.

#### 4.5.2. 3′ Adaptor Ligation

Since RNase A/T1 leaves a 3′ phosphate, RNA duplexes were treated with Shrimp Alkaline Phosphatase (rSAP) (NEB, Ipswich, MA, USA) for 30 min. After phenol/chloroform extraction and ethanol precipitation, RNA duplexes were ligated with adaptors as previously reported [23] with minor modification. The 3′ adaptor sequence was 5′-rApp/CTGTTAACNNNNNNNNNNNNNNNTGGAATTCTCGGGTGCCAAGGC/3ddC. The 3′ adaptor ligation was carried out at 25 °C for 2 h with a T4 RNA ligase 2 deletion mutant. The ligated products were separated on 15% acrylamide 8 M urea/TBE gels and eluted as described above.

#### 4.5.3. 5′ Adaptor Ligation

Eluted ligation products were phosphorylated using polynucleotide kinase (NEB, Ipswich, MA, USA) and 1 mM ATP for 30 min. After phenol/ chloroform extraction and ethanol precipitation, the products were used for 5′ adaptor ligation with T4 DNA ligase at 16 °C overnight. The 5′ adaptor sequence was 5′-AmMC6/CTACACGACGCTCTrGrUrUrArArCrArG, which is partially complementary with the 3′ adaptor to aid in ligation, and generates a Y-shaped structure in the final product. Ligated products were purified using phenol/chloroform extraction and ethanol precipitation.

#### 4.5.4. RT-PCR

After denaturing the ligated products for 3 min at 95 °C, reverse transcription was performed using ThermoScript™ Reverse Transcriptase (ThermoFisher Scientific, Waltham, MA, USA) at 60 °C for 1 h with a specific primer (CCTTGGCACCCGAGAATTCCA). The complementary DNA (cDNA) products were then amplified by PCR using Phusion High-Fidelity DNA polymerase (NEB) to generate a deep sequencing library. The reaction was carried out at 25 cycles of 98 °C for 10 s, 60 °C for 30 s, and 72 °C for 15 s using the 5′ end primer (AATGATACGGCGACCACCGAGATCTACACTCTTTCCCTACACGACGCTCTTCCGATCT) and the 3′ end primer (CAAGCAGAAGACGGCATACGAGATNNNNNNGTGACTGGAGTTCCTTGGCACCCGAGAATTCCA).

#### 4.5.5. Deep Sequencing

The PCR products were separated on 15% acrylamide TBE native gels. Sizes of DNA around 143 bp were sliced from gels and broken down by gel breaker tubes (IST Engineering). DNAs were eluted overnight at 4 °C, and gel debris were filtered out by 5-μm filter tubes (IST Engineering). Eluted DNAs were precipitated by ethanol. The concentration of DNA was measured with a Qubit fluorometer (ThermoFisher Scientific). The sizes of the DNAs were confirmed using an Agilent Technologies 2100 Bioanalyzer with High-Sensitivity DNA Kit (Agilent Technologies, Santa Clara, CA, USA). Sequencing was performed with Illumina MiSeq^®^ Reagent Kit v3 (150 cycles) (Illumina, San Diego, CA, USA) as per the manufacturer’s instruction on Illumina MiSeq.

### 4.6. Datasets

Sequencing data (in FASTQ format) was obtained for 15 datasets listed in Table S1 (Supplementary Materials). The datasets S1–S13 correspond to four different cell lines (293T, MCF7, Hela, and U2OS) some of which were subjected to three different treatments ((i) cell lysate, (ii) proteins removed, and (iii) both ribosomal RNA and proteins removed) as listed in Table S1 (Supplementary Materials). The two datasets S14 and S15 listed in Table S1 correspond to RNA extracted from the band that appears consistently in 293T and Hela cells, respectively (also indicated with an arrow in Figure 2A). One dataset corresponds to 293T native lysates with spiked-in 30 base pair RNA duplexes with 5-nt overhangs as a positive control (sequences A/B listed in Section 4, see also Table S1, Supplementary Materials). The datasets underwent a bioinformatics filtering step such that they consisted of reads with length greater than 18 nt. Note that, among the three mentioned treatments, only cell lysates (treatment (i)) corresponds to measured RNA duplexes in the presence of proteins, while both treatments (ii) and (iii) correspond to the removal of proteins via the extraction of RNA, followed by denaturing and reannealing of RNA.

The data discussed in this publication were deposited in the National Center for Biotechnology Information (NCBI)’s Gene Expression Omnibus (GEO) [38] and are accessible through GEO Series accession number GSE117821 (https://www.ncbi.nlm.nih.gov/geo/query/acc.cgi?acc=GSE117821). 

### 4.7. Data Analysis

Unless a specific tool is mentioned in the description of the data analysis, the R programming language was utilized for data processing.

### 4.8. Reference Data Set

Reference transcript sequences were compiled using four databases: (1) ENSEMBL (release 84, [39]), (2) LNCipedia v. 4.0 [40], (3) tRNAdb [41], and (4) RefSeq ([42], accessed May 27, 2016). Transcripts that were part of several underlying databases were added only once, giving precedence to the database according to the ordered list of databases mentioned above. This resulting dataset consisted of 304,552 transcript variants corresponding to 121,176 distinct genes.

### 4.9. Mapping of Reads to Transcripts

Reads were preprocessed in the form of a sliding window quality score filtering using the Sickle program with default options [43]. Reads were mapped to the set of 304,552 transcript sequences (described in the previous paragraph) using the alignment program Salmon [44]. Differential transcript expression with respect to the different treatments was quantified using the DESeq2 R package [45]. Because the Salmon program does not provide information about where on the transcript a read is matching, two additional alignment strategies were used in order to carry out additional analysis steps: Reads were mapped to human genome assembly hg38 using HISAT [1]. For subsequent analysis of which genome positions were needed (i.e., the analysis of sense/antisense enrichment), the HISAT mapping was utilized. Reads were additionally mapped to transcript sequences of the reference dataset described above using BLAST/BLASTn (version 2.2.26) (NCBI, NIH, Bethesda, MD, USA), followed by post-processing using R to require perfect sequence identity between the read sequence and the matching transcript sequence.

### 4.10. Search for Long Duplexes

The RNAduplex program was utilized to identify read-pairs with long reverse-complementary regions [46]. The program was applied to all reads of each dataset in an all-versus-all comparison of all reads of the same length. In other words, we performed secondary-structure predictions of potential read–read interactions of all the same-length read pairs that originate from the same dataset. For each read, the number of base pairs of the longest predicted helix with respect to all other read pairs was recorded.

### 4.11. Identifying Enrichment in Coverage

The coverage of reads with respect to the three different treatments was computed by computing the coverage for each transcript (how many reads map to a certain nucleotide position on its RNA sequence, taking into account a correction for multiple matches of reads onto the transcriptome). Next, the per-position read coverage was averaged over all positions of a transcript. The resulting per-transcript coverage values were averaged over all transcripts of a gene in order to compute per-gene average coverage values. Utilizing the gene type classification (based on the classification provided by the ENSEMBL database), one can, in this fashion, compute an average coverage value per gene class. For each gene class, the ratio of the average coverage for two treatments was used as enrichment (for example, the treatment “removal of proteins” versus treatment “native lysate”).

### 4.12. Search for Evidence of Endo-Ligases

We searched for chimeric reads that could be matched to two different transcripts in a manner that suggests that endo-ligase-ligated RNA fragments were located in close proximity, effectively performing an RNA proximity ligation experiment [7]. We required that matches between a read and the two transcripts be non-overlapping, at least 18 base pairs long, and either starting at the 5′ end of a read or ending with the 3′ end.

## 5. Conclusions

In conclusion, we present a novel experimental protocol coupled with computational analysis applied to several cell line datasets with genomic information about RNA–RNA interactions. We found evidence that RNA–RNA interactions were far less common than one would expect by simply analyzing transcriptome sequences. Also, RNA–RNA interactions were more ubiquitous when removing proteins from cell extracts. The diversity of results from different cell lines suggests that RNA–RNA interactions are to a large extent stochastic events. These findings have important implications for the design of therapeutic RNAs resulting in two strategies of either avoiding or purposefully introducing long RNA helices.

## Supplementary Materials

The following are available online. Table S1: List of data sets, Table S2: Enrichment of overlapping sense-antisense regions, Figure S1: Negative selection of RNAs with respect to ribosomal expansion segments, Figure S2: Depiction of ribosomal expansion segment regions.
