# Supplementary material for: RNA–Protein Interactions Prevent Long RNA Duplex Formation: Implications for the Design of RNA-Based Therapeutics
Source: Molecules. 2018 Dec 15;23(12):3329. doi: 10.3390/molecules23123329 (PMC6321275; doi:10.3390/molecules23123329)
Supplement: Supplementary file 1 [file molecules-23-03329-s001.pdf]

# Supplementary Information

## RNA–Protein Interactions Prevent Long RNA Duplex Formation: Implications for the Design of RNA-Based Therapeutics

Table S1: List of data sets
Table S2: Enrichment of overlapping sense-antisense regions
Figure S1: Negative selection of RNAs with respect to ribosomal expansion segments
Figure S2: Depiction of ribosomal expansion segment regions

| ID | Cell | Treatment |
|---|---|---|
| S1 | 293T | native with added control duplex |
| S2 | 293T | native |
| S3 | 293T | protein removed |
| S4 | Hela | native |
| S5 | Hela | protein removed |
| S6 | 293T | native |
| S7 | 293T | ribo removed |
| S8 | Hela | native |
| S9 | Hela | ribo removed |
| S10 | MCF7 | native |
| S11 | MCF7 | ribo removed |
| S12 | U2OS | native |
| S13 | U2OS | ribo removed |
| S14 | 293T | rRNA band |
| S15 | Hela | rRNA band |

**Table S1:** List of data sets corresponding to 4 cell lines and 3 treatments as described in the Materials and Methods section. Two data sets (S14 and S15) pertain to sequencing data corresponding to the bands shown in Figure 2A (indicated by an arrow).

| | C1 | C2 | C3 | C4 | C5 | C6 | C7 | C8 | C9 | C10 | C11 | C12 | C13 |
|---|---|---|---|---|---|---|---|---|---|---|---|---|---|
| ID | sas+exp+ | sas-exp+ | sas+exp- | sas-exp- | C1+C3 | C2+C4 | C1+C2 | C3+C4 | C1/(C1+C2) | C3/(C3+C4) | C1/(C1+C3) | C2/(C2+C4) | (C1/C1+C2))/(C 3/(C3+C4) |
| **S1** | 2180 | 62726 | 1386 | 87765 | 3566 | 150491 | 64906 | 89151 | 0.034 | 0.016 | 0.611 | 0.417 | 2.160 |
| **S2** | 997 | 63909 | 543 | 88608 | 1540 | 152517 | 64906 | 89151 | 0.015 | 0.006 | 0.647 | 0.419 | 2.522 |
| **S3** | 8273 | 56633 | 4861 | 84290 | 13134 | 140923 | 64906 | 89151 | 0.127 | 0.055 | 0.630 | 0.402 | 2.338 |
| **S4** | 734 | 64172 | 417 | 88734 | 1151 | 152906 | 64906 | 89151 | 0.011 | 0.005 | 0.638 | 0.420 | 2.418 |
| **S5** | 12030 | 52876 | 7375 | 81776 | 19405 | 134652 | 64906 | 89151 | 0.185 | 0.083 | 0.620 | 0.393 | 2.241 |
| **S6** | 3545 | 61361 | 2260 | 86891 | 5805 | 148252 | 64906 | 89151 | 0.055 | 0.025 | 0.611 | 0.414 | 2.155 |
| **S7** | 96 | 64810 | 54 | 89097 | 150 | 153907 | 64906 | 89151 | 0.001 | 0.001 | 0.640 | 0.421 | 2.442 |
| **S8** | 1650 | 63256 | 996 | 88155 | 2646 | 151411 | 64906 | 89151 | 0.025 | 0.011 | 0.624 | 0.418 | 2.275 |
| **S9** | 238 | 64668 | 98 | 89053 | 336 | 153721 | 64906 | 89151 | 0.004 | 0.001 | 0.708 | 0.421 | 3.336 |
| **S10** | 3285 | 61621 | 1919 | 87232 | 5204 | 148853 | 64906 | 89151 | 0.051 | 0.022 | 0.631 | 0.414 | 2.351 |
| **S11** | 1615 | 63291 | 857 | 88294 | 2472 | 151585 | 64906 | 89151 | 0.025 | 0.010 | 0.653 | 0.418 | 2.588 |
| **S12** | 1274 | 63632 | 836 | 88315 | 2110 | 151947 | 64906 | 89151 | 0.020 | 0.009 | 0.604 | 0.419 | 2.093 |
| **S13** | 959 | 63947 | 486 | 88665 | 1445 | 152612 | 64906 | 89151 | 0.015 | 0.005 | 0.664 | 0.419 | 2.710 |

**Table S2:** Transcripts are more likely to be part of the experimental data if they have genomic overlap with an antisense transcript (indicated as sas+). The rows 1-13 correspond to data sets S1 to S13 listed in Table S1. C1:sas+exp+: sense-antisense regions found in data; C2:sas-exp+: transcripts without sense-antisense regions found in data; C3:sas+exp-: transcripts with sense-antisense regions not found in data; C4:sas-exp-: transcripts without sense-antisense regions not found in data. Column 13 is the odds ratio of column C9 and C10 and indicates that transcripts are more than 2 times more likely to be part of the experimental data if their genomic regions overlap an antisense transcript.

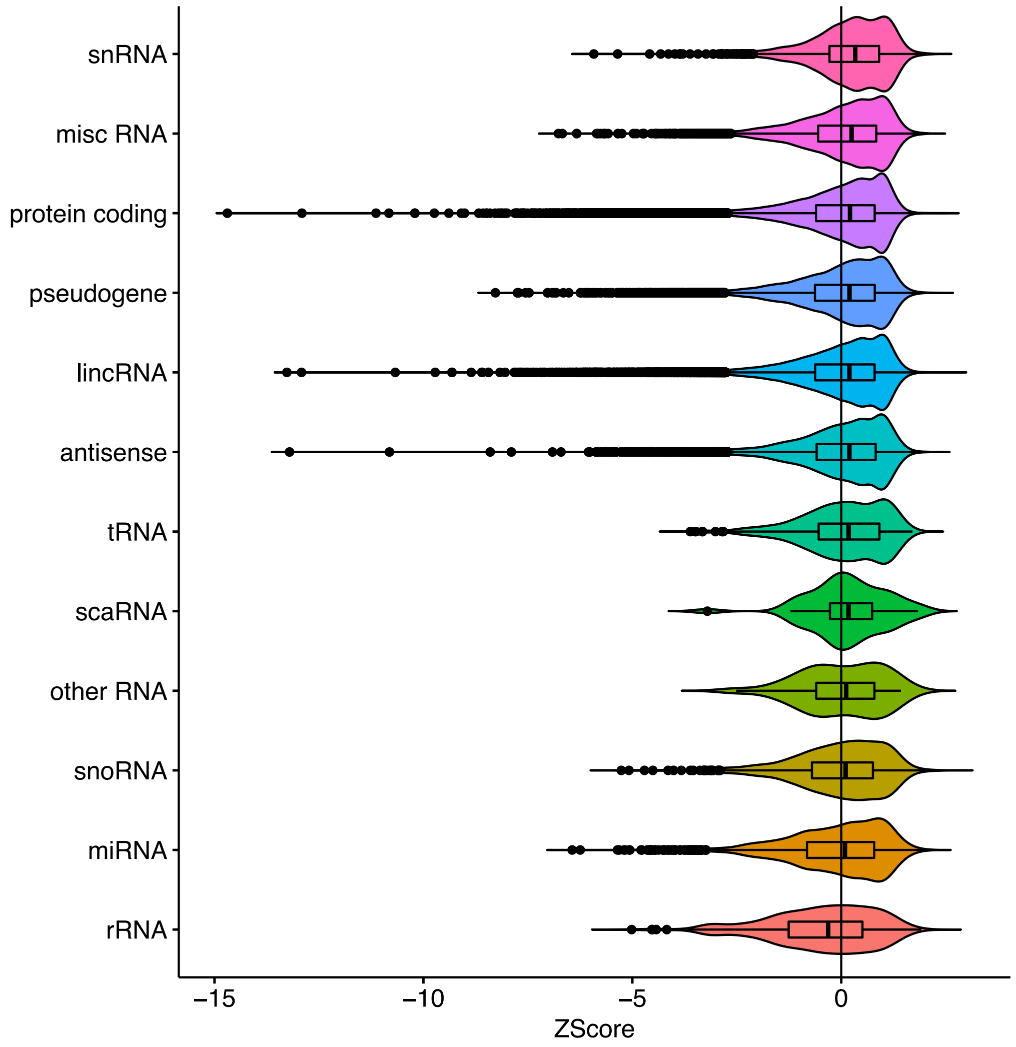


**Figure S1: Negative selection of RNAs with respect to expansion segment 27 of the 28S large ribosomal subunit.** For most RNA classes (with exception of rRNA), the computed interaction energy (based on the RNAduplex program) is higher than expected by chance (ascertained by computing interaction energies for 20 shuffled control sequences).

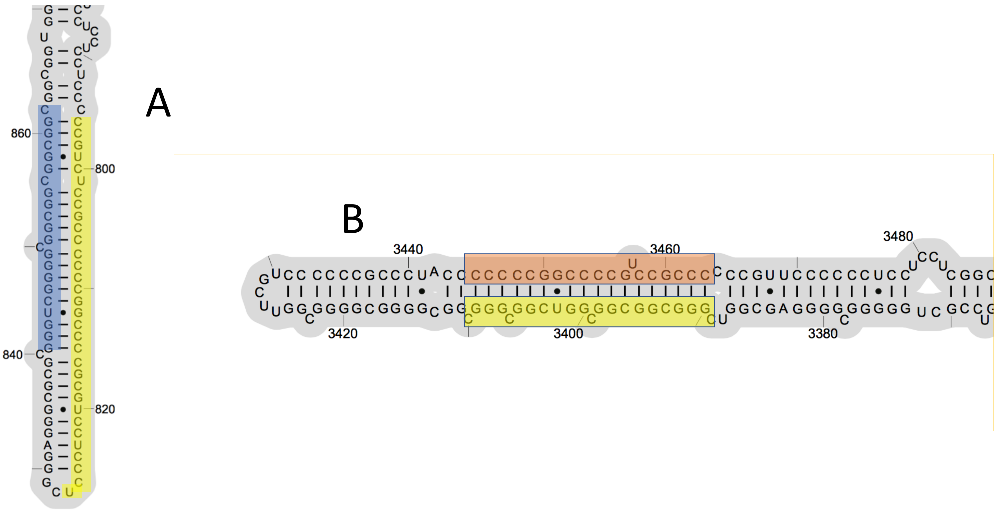


**Figure S2.** Part of expansion region (A) ES7L and (B) ES27L of human 28S ribosomal RNA. Shown in yellow (positions 796-827 for A and 3389-3410 for B) are regions that correspond to highly prevalent reads in the sequencing data. The coverage of the ES7L region shown in blue (positions 841-862) is lower but still higher than the regions shown in grey. The coverage of the ES27L region shown in orange (positions 3445-3463) is dramatically lower. Adapted from (Anger et al., *Nature*, 2013).
